# N6-methyladenosine modification in the context of viral infection: from molecular mechanism to therapeutic potential

**DOI:** 10.1016/j.virusres.2026.199749

**Published:** 2026-05-19

**Authors:** Xinzhe Wu, Zhuoxuan Deng, Yunkai Li, Chunwei Shi

**Affiliations:** Department of Pathogen Biology, School of Basic Medicine, Tongji Medical College, Huazhong University of Science and Technology, Wuhan, PR China

**Keywords:** N^6^-methyladenosine, Virus, Epitranscriptome, Antiviral strategy

## Abstract

N^6^-methyladenosine (m^6^A) is the most abundant and dynamically regulated RNA modification in eukaryotic cells, which modulates key RNA metabolic processes including splicing, stability, nuclear export, degradation and translation. During viral infection, m^6^A exerts a pivotal regulatory role by modifying both viral RNAs and host cellular transcripts. Accumulating evidence demonstrates that viruses hijack the host m^6^A machinery through diverse mechanisms to reprogram the host epitranscriptome, in favor of viral replication. Targeting the host m^6^A machinery has emerged as a promising novel antiviral therapeutic strategy. This review focuses on the molecular mechanisms underlying viral hijacking of host m^6^A modifications and explores the therapeutic potential of targeting the viral hijacking mechanisms of the host m^6^A machinery.

## Highlights

• The m⁶A machinery dynamically modulates multiple RNA metabolic processes and regulates viral infection.
• Viruses hijack host m⁶A machinery to reshape cell epitranscriptome and facilitate viral proliferation.
• Targeting the mechanisms of viral hijacking m⁶A machinery provides a promising direction for antiviral therapy

RNA modifications in eukaryotic cells serve as critical regulators of post-transcriptional gene expression. They are widely distributed across mRNA, tRNA, rRNA and diverse non-coding RNAs, constituting an additional regulatory layer beyond DNA and histone modifications (Roundtree et al., 2017; Cui et al., 2022). By modulating RNA metabolism and gene expression, RNA modifications maintain cellular homeostasis and exert pivotal roles in the pathogenesis of various diseases, including viral infections (Thompson et al., 2021). N6-methyladenosine (m^6^A), 5-methylcytosine (m^5^C), N1-methyladenosine (m^1^A), N7-methylguanosine (m^7^G), and 2’-O-methylation (Nm) represent the most prevalent RNA methylation modifications in mammalian cells (Liu et al., 2024; Arzumanian et al., 2022).

Viruses can reshape RNA modifications via multiple strategies. One mechanism of metabolic reprogramming is to indirectly modulate RNA modifications by altering upstream epitranscriptomic substrates. For instance, coronaviruses including SARS-CoV-2 have been shown to activate the mTORC1 signaling pathway, resulting in elevated MAT2A expression and increased intracellular levels of S-adenosylmethionine (SAM), which globally enhances m^6^A deposition and facilitates viral replication (Zhou et al., 2025). Alternatively, viruses can employ a more direct strategy, whereby viral factors hijack host RNA modification machineries through molecular interactions and modulate their abundance, subcellular localization or catalytic activity.

Among all RNA modifications, m^6^A modification is the most extensively characterized. m^6^A deposition is a reversible process governed by writers, erasers and readers. The m^6^A writer complex comprises the METTL3-METTL14 heterodimer as its catalytic core, together with several auxiliary RNA-binding regulatory subunits (Liu et al., 2014; Wang et al., 2016). The eraser proteins FTO and ALKBH5 remove m^6^A via oxidative demethylation, whereas m^6^A readers, mainly members of the YTH family, recognize methylated RNAs and modulate their post-transcriptional processing, stability, translation and degradation (Jia et al., 2011; Zheng et al., 2013; Yang et al., 2018). Accordingly, the m^6^A regulatory machinery serves as a key hub in virus-host interactions.

In this review, we first summarize the major mechanisms of host RNA modifications, then focuses primarily on m^6^A as a paradigm to illustrate how viruses exploit the host RNA methylation machinery to facilitate their life cycles. We further discuss emerging antiviral strategies targeting both host m^6^A machinery and viral hijacking mechanisms, aiming to provide a new perspective for the development of antiviral therapies at epitranscriptomic level.

## 1 RNA modifications and their roles in viral infection

Eukaryotic RNAs harbor a variety of chemical modifications that regulate RNA processing, stability, localization and translation (Roundtree et al., 2017). Collectively, these modifications expand the regulatory potential of the transcriptome and contribute to diverse biological processes under both physiological and pathological conditions (Roundtree et al., 2017; Cui et al., 2022). Among these modifications, m^5^C, m^7^G, 2’-O-methylation and m^6^A are the extensively characterized RNA methylation mechanisms and have been increasingly implicated in host responses to viral infection (Thompson et al., 2021; Arzumanian et al., 2022). m^5^C is an important RNA modification mediated by NSUN (NOP2/Sun RNA methyltransferase) family members (Yang et al., 2017). It promotes mRNA export via ALYREF (Aly/REF export factor) and enhances transcript stability through binding to YBX1 (Y-box binding protein 1), thereby modulating gene expression during development, tumorigenesis and viral infection (Yang et al., 2017; Chen et al., 2019). M^7^G modification is best known as a defining feature of the 5’-cap structure, where it is installed by the RNMT-RAM complex and recognized by cap-binding proteins such as eIF4E (eukaryotic translation initiation factor 4E) to facilitate mRNA stability and translation initiation (Ramanathan et al., 2016; Sonenberg and Hinnebusch, 2009; Gonatopoulos-Pournatzis et al., 2011). Additionally, internal m^7^G modifications are installed by the METTL1-WDR4 complex, which can enhance the translation of specific transcripts. In particular, METTL1/WDR4-mediated tRNA m^7^G modification has been implicated in tumorigenesis by promoting oncogenic translation (Zhang et al., 2019).

Another important modification is 2’-O-methylation of the mRNA cap, which serves as a molecular signature for self RNA (Züst et al., 2011). RNAs lacking this modification are preferentially bound by IFIT1 (Interferon-Induced Protein with Tetratricopeptide Repeats 1), resulting in translational inhibition and restriction of viral replication (Habjan et al., 2013; Abbas et al., 2017). To evade immune detection, many viruses encode their own 2’-O-methyltransferases to mimic host cap structures (Abbas et al., 2017; Daffis et al., 2010; Szretter et al., 2012).

Among internal RNA modifications in eukaryotic mRNA, m^6^A is the most abundant and most extensively studied (Dominissini et al., 2012; Meyer et al., 2012), playing critical roles in cell differentiation, embryonic development and disease pathogenesis (Thompson et al., 2021; Liu et al., 2014).

These RNA modifications are dynamically regulated during viral infection. In virus-infected cells, the cellular RNA methylation machinery modulates both host and viral RNAs. Accumulating evidence indicates that viral infection can profoundly reshape host RNA modifications, reprogramming host RNA metabolism to promote viral replication and suppress antiviral immunity. Epitranscriptomic reprogramming mediated by RNA modifications has been reported in multiple RNA and DNA viruses, including HIV-1 (Human immunodeficiency virus type 1), HBV (Hepatitis B virus), EBV (Epstein–Barr virus), KSHV (Kaposi’s sarcoma-associated herpesvirus) and HCV (Hepatitis C virus) (Table 1)**.**

**Table 1:** Viral regulation of host epitranscriptomic RNA modification.

Virus	Modification	Site	Function	Reference
Flaviviridae (IKV, DENV, WNV)	m^6^A	mRNA	Alters the m^6^A modification of specific host transcripts (such as RIOK3 and CIRBP) to promote viral infection	Gokhale et al., 2020
DENV	2’-O-Me m^1^A	tRNA	Reprogramms the host tRNA epitranscriptome to promote the translation of proteins required for viral replication	Zou et al., 2024
JEV	m^5^C	mRNA	Increases m^5^C modification of Cebpd mRNA and accelerates its degradation to promote viral persistence by suppressing cGAS-STING-dependent innate immune signaling	Chen et al., 2025
HCV	m^5^C	mRNA	Upregulates the host m^5^C methyltransferase NSUN2 to increase m^5^C modification on host mRNAs, suppressing host innate antiviral immune responses	Li et al., 2025
SARS-CoV-2	m^6^A	mRNA or lncRNA	Reprograms host m^6^A epitranscriptome by globally reducing m^6^A on cellular RNAs, while selectively remodeling m⁶A on immune-related lncRNAs to attenuate antiviral stress responses	Peter et al., 2025, Vaid et al., 2023
	m^5^C	mRNA	Increases m^5^C modification on IRF3 mRNA and promotes type I interferon and downstream ISG expression	Wang et al., 2023
HIV-1	m^6^A	mRNA	Induces widespread hypermethylation of host mRNAs via m^6^A to promote HIV-1 gene expression and replication	Lichinchi et al., 2016b, Mishra et al., 2024, Peng et al., 2023, Ramanathan et al., 2016
	m^5^C	mRNA	Induces hypermethylation of host mRNAs via m^5^C, preferentially targeting transcripts involved in HIV replication and host gene regulation	Ramanathan et al., 2016
KSHV	m^6^A/m^6^Am	mRNA	Reprograms host m^6^A/m^6^Am epitranscriptome in a phase- and cell type-dependent manner, redistributing m^6^A from host to viral transcripts and modulating RNA stability, decay and oncogenic signaling pathways to regulate latency maintenance and lytic replication	Hesser et al., 2018, Macveigh-Fierro et al., 2022, Tan et al., 2018
EV71	m^6^A	mRNA	Increases m^6^A modification of immune- and inflammation-related mRNAs to promote inflammasome activation, NF-κB signaling and viral replication	Hu et al., 2023, Liu et al., 2025, Peng et al., 2025
HBV	m^6^A	mRNA	Increases m^6^A modification of host PTEN mRNA, leading to reduced PTEN mRNA stability and protein expression, thereby suppressing IRF3 dependent interferon production and activating PI3K/AKT signaling to promote immune evasion and hepatocarcinogenesis	Kim et al., 2021

## 2 m^6^A machinery

m^6^A is enriched in the consensus RRACH motif, predominantly distributed around stop codons within 3’-untranslated regions (3’-UTRs), and across long internal exons (Meyer et al., 2012). The dynamic m⁶A machinery is governed by methyltransferases, demethylases and m^6^A-binding proteins, which are collectively referred to as “writers”, “erasers” and “readers” (Liu and Pan, 2016; Edupuganti et al., 2017; Jiang et al., 2021). Notably, the role of m^6^A in viral infections has attracted increasing attention. m^6^A modification regulates viral infections by modifying either viral RNAs or host cellular transcripts, thereby exerting pro-viral or anti-viral effects (Zhang et al., 2022; Horner and Reaves, 2024).

### 2.1 The writer of m^6^A machinery: m⁶A methyltransferase complex

**Fig. 1:**
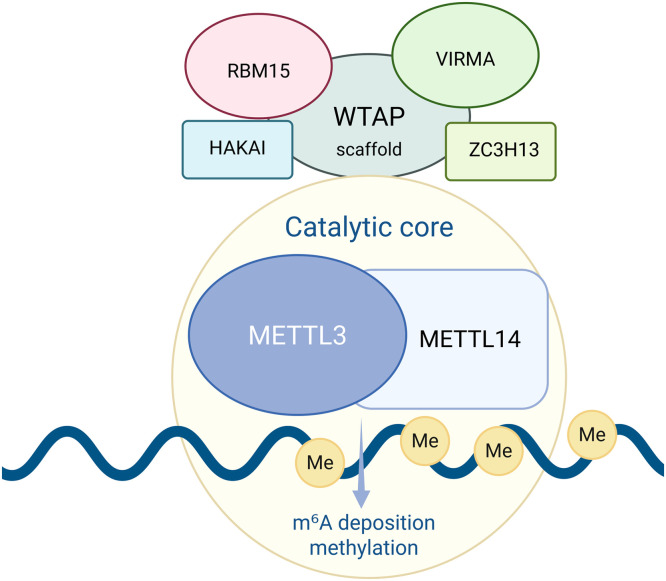
The m^6^A writer complex and its regulatory components. Schematic overview of the m^6^A writer machinery, in which METTL3 and METTL14 constitute the catalytic core mediating m^6^A methylation, while WTAP serves as a scaffold protein. RBM15, VIRMA, HAKAI and ZC3H13 are accessory factors that coordinate complex stability, target recruitment and efficient m^6^A deposition on RNA substrates.

The m^6^A writer is a multi-component complex, whose catalytic core consists of a METTL3-METTL14 heterodimer (Fig. 1). METTL3 acts as the catalytically active subunit, containing the SAM-binding site and the DPPW (Asp-Pro-Pro-Trp) catalytic motif, while METTL14 plays a structural role in supporting METTL3 function (Liu et al., 2014; Wang et al., 2016). Other auxiliary proteins of the writer complex are reported to be indispensable for m^6^A modification (Pandey et al., 2025). Specifically, WTAP facilitates the localization of the METTL3-METTL14 heterodimer to nuclear compartments and serves as a scaffold of the writer complex binding with HAKAI, ZC3H13, VIRMA and RBM15 (Fig. 1) (Liu et al., 2024; Arzumanian et al., 2022; Zhou et al., 2025), which mediate methylation specificity. VIRMA recruits METTL3 to the 3’-end of mRNAs, while RBM15 binds to U-rich sequences adjacent to m^6^A consensus sites, facilitating methylation in a WTAP-dependent manner (Patil et al., 2016; Ping et al., 2014; Morshed et al., 2025; Schwartz et al., 2014). Although the specific functions of HAKAI and ZC3H13 remain to be elucidated, both have been shown to be essential for the proper function of the m^6^A writer complex (Bawankar et al., 2021; Pinto et al., 2020). Notably, METTL16 and METTL5 act as specific methyltransferases for m^6^A modification of U6 spliceosomal snRNA and 18S rRNA, respectively (Pendleton et al., 2017; Turkalj and Vissers, 2022).

### 2.2 The eraser of m^6^A machinery

Currently, FTO and ALKBH5, have been reported to function as demethylases and catalyze the removal of m^6^A modifications, enabling the reversible regulation of this RNA mark (Jia et al., 2011; Zheng et al., 2013). However, Mauer et al. reported that FTO exhibits a preference for demethylating m^6^Am rather than m^6^A (Mauer et al., 2017). The existence of other demethylases, as well as the detailed molecular mechanisms underlying these demethylases, remain to be further explored.

### 2.3 The reader of m^6^A machinery

As the earliest identified m^6^A readers, YT521-B homology (YTH) domain-containing proteins (YTHDF1-3, YTHDC1-2) function cooperatively to exert a wide range of effects on RNA metabolism (Stoilov et al., 2002). YTHDF1 promotes mRNA translation by interacting with the translation machinery, while YTHDF2 mediates the degradation of its target mRNAs by recruiting degradation machinery, such as the CCR4-NOT deadenylase complex and RNase P/MRP complex, through its N-terminal domain (Park et al., 2019; Wang et al., 2014; Du et al., 2016; Wang et al., 2015). YTHDF3 modulates the accumulation of cellular mRNAs by interacting with YTHDF1 and YTHDF2 respectively (Shi et al., 2017). YTHDC1 mediates pre-mRNA splicing by recruiting the splicing activator SRSF3 (Serine and Arginine Rich Splicing Factor 3) and excluding the repressor SRSF10 (Serine and Arginine Rich Splicing Factor 10) from target transcripts, whereas YTHDC2 resolves mRNA secondary structures to promote translation (Mao et al., 2019; Xiao et al., 2016). Additionally, HNRNPs (heterogeneous nuclear ribonucleoprotein) and IGF2BPs (Insulin-like Growth Factor 2 mRNA-Binding Proteins) regulate RNA metabolism in an m^6^A-dependent manner. HNRNPs stabilize target mRNAs, while IGF2BPs modulate microRNA processing and splicing (Huang et al., 2018; Alarcón et al., 2015). The identification of writers, erasers and readers underscores the dynamic and reversible nature of m^6^A-mediated post-transcriptional regulation (Zou et al., 2024).

## 3 Molecular regulation of host m^6^A modification by viruses

### 3.1 Degradation of m^6^A molecules by ubiquitin-proteasome system of host cells

Ubiquitination is a critical post-translational modification that labels proteins for degradation via the proteasomal system, collectively referred to the ubiquitin-proteasome system (UPS) (Pickart, 2001). Multiple viruses have been reported to hijack E3 ubiquitin ligases through their non-structural proteins, thereby specifically targeting m^6^A writers for proteasomal degradation via host UPS. For instance, when EBV (Epstein-Barr virus) infects B cells, EBV-encoded protein EBNA1 (Epstein–Barr Nuclear Antigen 1) upregulates the host E3 ubiquitin ligase Parkin, which promotes K48-linked polyubiquitination of METTL3 and facilitates its proteasomal degradation, leading to a global reduction in cellular m^6^A levels. Consequently, m^6^A modification on Toll-like receptor 9 (TLR9) mRNA is decreased, resulting in reduced mRNA stability and expression, thereby suppressing TLR9-mediated antitumor immune responses (Zhang et al., 2024). In infected keratinocytes, The high‑risk HPV (Human papillomavirus) E6 protein interacts with the host E3 ubiquitin ligase E6AP to induce proteasomal degradation of METTL3 and METTL14, resulting in a global decrease of m^6^A modification. Reduced m^6^A deposition stabilizes RNA:DNA hybrids (R-loops) and promotes R-loop accumulation, which is critical for HPV genome replication and transcription (Templeton and Laimins, 2026). Additionally, SARS-CoV-2 (Severe Acute Respiratory Syndrome Coronavirus 2) has also been reported to interact with METTL3 and inhibit its ubiquitination and SUMOylating through an unknown mechanism, thereby promoting m^6^A modification of viral RNA and facilitating viral replication (Zhang et al., 2021).

In addition to hijacking host E3 ubiquitin ligases, some viruses encode their own E3 ubiquitin ligases to directly target m^6^A writers. For example, HSV-1 (Herpes simplex virus 1) has been reported to utilize its RING-finger E3 ubiquitin ligase ICP0 in U87MG and D54 glioma cells to ubiquitinate and degrade METTL14, thereby reducing m^6^A-dependent stabilization of ISG15 (Interferon-Stimulated Gene 15) mRNA and impairing the host antiviral response (Chen et al., 2024).

**Fig. 2:**
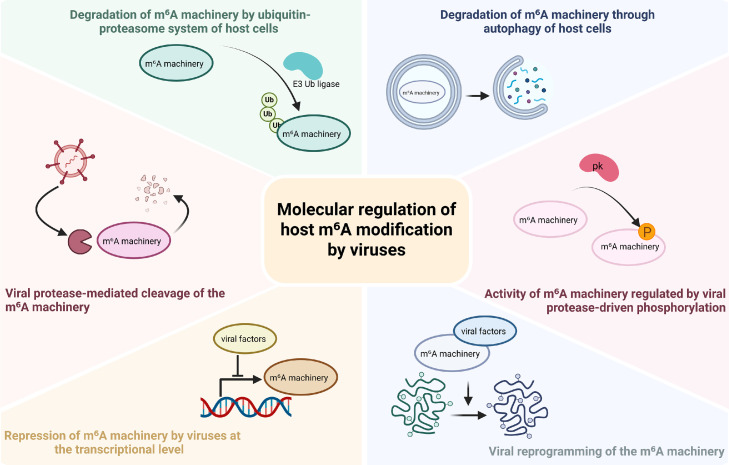
Viral Modulation of the Host m^6^A Machinery. Viral factors can induce the ubiquitin-proteasome dependent degradation, autophagy-mediated turnover or protease-dependent cleavage of m^6^A regulators. Viruses can also exert control at the transcriptional level, induce post-translational modifications or reprogram the function of the m^6^A machinery. Collectively, these processes orchestrate changes in the abundance, subcellular localization or activity of m^6^A writers, readers and erasers. Such alterations ultimately reshape the fate of m^6^A-modified RNAs, thereby regulating viral replication, antiviral signaling and immune evasion.

Beyond inducing ubiquitin-mediated degradation of host m^6^A machinery, viruses may also manipulate the ubiquitin machinery to prevent the degradation of selected host proteins. Specifically, the NS5B (Nonstructural Protein 5B) protein of CSFV (Classical swine fever virus) sequesters the E3 ubiquitin ligase HRD1 in the cytoplasm of PK-15, IPEC-J2 and ST cells, thereby blocking HRD1-mediated K27-linked ubiquitination of METTL14 and resulting in METTL14 stabilization. Elevated METTL14 enhances m^6^A modification within the 3’-UTR of TLR4 (Toll-like receptor 4) mRNA, which in turn promotes YTHDF2-dependent mRNA decay, attenuates NF-κB signaling and facilitates immune evasion (Chen et al., 2024). Overall, ubiquitination serves as a critical mechanism by which viruses reprogram the m^6^A machinery to facilitate viral infection (Fig. 2).

### 3.2 Degradation of m^6^A molecules through autophagy of host cells

In addition to ubiquitin-proteasome system, viruses can also manipulate autophagy-dependent pathways to control the stability of m^6^A machinery (Fig. 2). Many viral proteins exploit the host autophagy machinery to degrade cellular proteins and further regulate viral replication (Gong et al., 2022; Sun et al., 2022). Viruses also utilize autophagy to degrade m^6^A molecules, thereby impairing m^6^A-regulated antiviral immunity (Liu et al., 2024). Specifically, the structural protein VP1 of FMDV (Foot-and-mouth disease virus) suppresses AKT-mTOR signaling to induce autophagy, which leads to selective autophagic degradation of YTHDF2 in infected PK-15 cells. The loss of YTHDF2 enhances the stability of GTPBP4 (GTP binding protein 4) mRNA, resulting in increased GTPBP4 protein level. The elevated GTPBP4 inhibits the binding of IRF3 (interferon regulatory factor 3) to the promoter of IFNB (interferon beta), thereby suppressing type I interferon production induced by FMDV (Liu et al., 2024). There are currently limited studies on the degradation of m^6^A proteins via host autophagy induced by viruses that cause human disease, and more research is needed to clarify the detailed mechanisms.

### 3.3 Viral protease-mediated cleavage of the m^6^A molecules

The roles of m^6^A readers in viral infection are diverse and highly context-dependent. For example, m^6^A readers have been reported to exert pro-viral effects during IAV (influenza A virus) and SARS-CoV-2 infection, but antiviral effects during EBV, RSV (respiratory syncytial virus) and HIV-1 infection (Liu et al., 2024; Burgess et al., 2021; Picavet et al., 2024; Jurczyszak et al., 2020). In viral infections where m⁶A readers function in an antiviral manner, viruses have evolved to utilize viral protease-mediated cleavage of m^6^A readers, thereby disrupting the antiviral defense mechanisms mediated by these m^6^A readers (Fig. 2).

A representative example of this mechanism is Enterovirus. YTHDF proteins act as positive regulators of type I interferon-induced JAK/STAT1 signaling. 2A protease of Enterovirus cleaves YTHDF family proteins early during infection by recognizing conserved LTTY’G motifs, thereby disrupting this antiviral response (Kastan et al., 2021). Additionally, YTHDF proteins promote the formation of stress granules (SG), which sequester viral 3D polymerase and dsRNA (double-stranded RNA) within the granules and prevent their involvement in viral translation during the early stages of replication. Coxsackievirus B3 protein was also reported to induce the cleavage of YTHDF proteins, thus promoting viral replication (Zhao et al., 2024).

YTHDF proteins have also been reported to act as restriction factors for HIV-1, reducing the availability of viral RNA for reverse transcription by binding to m⁶A-modified HIV-1 RNA (Lu et al., 2018). Specifically, YTHDF3 is incorporated into newly assembled HIV particles in a Gag-dependent manner and suppresses reverse transcription during the next infection cycle by binding to m^6^A-modified regions of the HIV RNA genome. However, during virion maturation, HIV protease cleaves virion-incorporated YTHDF3, abrogating the antiviral activity of YTHDF3 and ensuring efficient reverse transcription of mature virions (Jurczyszak et al., 2020).

### 3.4 Activity of m^6^A machinery regulated by viral protease-driven phosphorylation

A distinct strategy evolved by alpha herpesviruses involves the reprogramming of the host epitranscriptome through phosphorylation-dependent modulation of m^6^A writers, which is mediated by the US3 protein kinase encoded by alpha herpesvirus. In CEF (Chicken Embryonic Fibroblast) cells, the US3 protein encoded by MDV (Marek's Disease Virus) directly interacts with WTAP in the nucleus and phosphorylates specific serine residues (S273, S305, S314, and S375) within its C-terminal domain, which is reported to significantly reduce global m⁶A levels and suppress host protein translation (Wang et al., 2025). In ST cells infected by PRV (Pseudorabies Virus) and HSV-1, US3 induces phosphorylation of key m^6^A writer components, including METTL3, METTL14 and WTAP, which leads to dissociation of the m^6^A writer complex from chromatin and consequently results in a loss of m^6^A modification in host mRNA. Interestingly, the phosphorylation event is dependent on US3 expression but independent of its intrinsic kinase activity, suggesting that US3 might recruit or activate host kinases to promote this process (Jansens et al., 2022).

Another protein kinase of alpha herpesviruses, UL13 protein kinase, has been reported to phosphorylate FTO during PRV infection. This phosphorylation mediates the suppression of ISG expression and weakens the antiviral response, though this effect is likely independent of demethylase activity of FTO (Verhamme et al., 2025). Therefore, the phosphorylation of m^6^A proteins driven by viral proteases, which in turn affects the activity of m^6^A proteins, is also an important mechanism by which viruses regulate the epitranscriptomics of host cell (Fig. 2).

### 3.5 Repression of m^6^A machinery by viruses at the transcriptional level

Rather than altering the stability or enzymatic activity of m^6^A components, EBV controls the expression of m^6^A writers at the transcriptional level. BZLF1, EBV immediate-early protein, suppresses METTL3 transcription by binding to its promoter in a manner dependent on its dimerization domain and DNA-binding activity. Downregulation of METTL3 reduces m⁶A modification of KLF4 (Krüppel-like factor 4) mRNA, which decreases YTHDF2-mediated decay and enhances KLF4 mRNA stability. The upregulated KLF4 expression consequently promotes BZLF1 transcription and EBV lytic replication, forming a positive feedback loop that facilitates viral infection (Dai et al., 2021). Other viruses, including SARS-CoV-2, EV71 (Enterovirus 71) and PRV, have also been reported to downregulate the expression of the m^6^A components at the transcriptional level, though the underlying mechanisms remain to be elucidated (Fig. 2) (Hao et al., 2019; Li et al., 2021; Hubacek et al., 2024; Wang et al., 2025).

### 3.6 Viral reprogramming of the m⁶A machinery

Numerous studies have demonstrated that viral infections can induce widespread redistribution of m^6^A deposition landscapes across both viral and host transcriptomes, including HSV, KSHV, flavivirus and SARS-CoV-2 (Fig. 2) (Gokhale et al., 2020; Peter et al., 2025; Tan et al., 2018; Srinivas et al., 2021). Specifically, in HSV infected NHDF cells (normal human dermal fibroblasts), HSV ICP27 protein alters the localization of m^6^A writers, including METTL3, METTL14 and other regulatory proteins, leading to spatial re-organization of the m^6^A machinery and extensive changes in the m^6^A modification landscape (Srinivas et al., 2021). Through direct interaction with m^6^A machinery, viral components reprogram the m^6^A machinery through globally remodeling the cellular m^6^A landscape, selectively redirecting m^6^A deposition, or altering RNA processing outcomes.

One strategy of m^6^A reprogramming employed by HBV involves the direct recruitment of the m^6^A writers to viral transcripts. The viral protein HBx associates with the METTL3/METTL14 methyltransferase complex and recruits it to the HBV minichromosome, thereby increasing m^6^A deposition on viral transcripts. Additionally, HBx has been reported to facilitate the recruitment of the m^6^A writer complex to the PTEN gene locus, which is associated with increased m^6^A modification and reduced stability of PTEN transcripts (Kim and Siddiqui, 2021). Similarly, in PoRV (Porcine Reproductive and Respiratory Syndrome Virus) infection, the VP6 protein binds to METTL3 and drives its re-localization to the cytoplasm, redirecting the host writer complex toward viral RNA methylation and enhancing viral replication (Liang et al., 2025).

Additionally, viruses can reprogram the host m^6^A machinery by regulating RNA splicing. The NS1 protein of IAV interacts with core components of the m^6^A writer complex, METTL3 and METTL14. At low expression levels, NS1 promotes m^6^A deposition on viral RNA. While at high expression levels, it reduces the binding of METTL3 to NS mRNA, thereby decreasing m^6^A levels and establishing a negative feedback loop (Liao et al., 2025). The m^6^A mark at position A385 is recognized by the reader YTHDC1, whose binding creates steric hindrance that interferes with the association of the adjacent splicing factor SRSF3, thereby suppressing NS mRNA splicing. Through this mechanism, NS1 regulates the NEP/NS1 protein ratio to maintain the balance required for efficient viral replication (Liao et al., 2025).

Interestingly, viruses can also reprogram m^6^A machinery through upstream epigenetic pathways. During SARS-CoV-2 infection, the ORF8 protein upregulates the histone methyltransferase G9a via histone mimicry mechanism, promoting the interaction of G9a with METTL3 and translation of specific host “poised mRNAs” (Muneer et al., 2025). These poised mRNAs encode key pathological factors, including viral receptors (such as ACE2), pro-inflammatory cytokines, coagulation-related proteins, angiogenic factors and fibrosis markers. Additionally, G9a enhances m^6^A modification of viral RNA and facilitates its translation, thereby promoting viral replication while suppressing the host innate antiviral response (Muneer et al., 2025).

## 4 Development of antiviral drugs targeting viral hijacking of m^6^A machinery

### 4.1 Antiviral drugs targeting host m^6^A machinery

Targeting the m^6^A machinery represents a potential therapeutic avenue for antiviral intervention. DAA (3-deazaadenosine), which broadly reduces m^6^A methylation by inhibiting SAM hydrolase, has been reported to significantly suppress the replication of a wide spectrum of viruses (Zou et al., 2024; Feng et al., 2021; Yanagi et al., 2022). However, as a nonspecific inhibitor of m^6^A, DAA is primarily utilized as a research tool to validate the function of the m^6^A pathway in viral infection due to its substantial off-target effects and challenges in toxicity control. Drugs that more precisely target the m^6^A machinery hold greater promise for clinical application.

#### 4.1.1 Antiviral drugs targeting m^6^A writers

Among the components of the m^6^A machinery, the m^6^A writer complex, particularly the catalytic subunit METTL3, has emerged as a central target for host-directed antiviral intervention. STM2457, which inhibits METTL3 activity by occupying its SAM-binding pocket, has been shown to significantly suppress the replication of SARS-CoV-2 and HCoV-OC43 (human coronavirus OC43) in vitro (Liu et al., 2024; Li and Gregory, 2021). Moreover, STM2457 is highly specific for METTL3 and exhibits no inhibition of other RNA methyltransferases, which minimizes off-target effects (Yankova et al., 2021; Fiorentino et al., 2023). Another METTL3 inhibitor, UZH1a, has been reported to inhibit the lytic replication of EBV, through binding with the SAM site of the METTL3-METTL14 complex and competitively impeding its methyltransferase activity (Yanagi et al., 2022; Li and Gregory, 2021). Other METTL3 inhibitors, such as EP652, which exerts anti-tumor effects, and Coptisine chloride, which has been proven to inhibit the progression of periodontitis, remain to be further investigated for their antiviral potential (Dutheuil et al., 2025; Zhou et al., 2024).

While inhibition of m^6^A modification has emerged as a potential strategy to suppress HIV replication, enhancing m^6^A methylation may also be exploited within the framework of the “shock-and-kill” strategy. This approach aims to reactivate latent HIV reservoirs (“shock”), thereby inducing viral transcription and protein expression in previously silent infected cells, followed by immune-mediated clearance or viral cytopathic effects (“kill”). Small-molecule METTL3/METTL14/WTAP activator compounds have been developed and shown to enhance m^6^A modification and HIV-1 replication in vitro, whose potential clinical application remains to be fully evaluated (Table 2) (Selberg et al., 2021).

**Table 2:** Small-molecule activators of the METTL3/METTL14/WTAP complex and their effects on HIV-1 replication.

**Chemical Name**	**Mechanism**	**Effect**	**Reference**
Tert-butyl 6-methylpiperidine-3-carboxylate	Activates m^6^A writer complex	Promotes viral particle production, without increased infectivity	Selberg et al., 2021
Methyl 6-methylpiperidine-3-carboxylate	Activates m^6^A writer complex	Promotes viral particle production and infectivity	Selberg et al., 2021
Methyl piperazine-2-carboxylate	Activates m^6^A writer complex	Promotes viral particle production and infectivity	Selberg et al., 2021
Ethyl 2-oxopiperidine-3-carboxylate	Activates m^6^A writer complex	Promotes viral particle production and infectivity	Selberg et al., 2021

#### 4.1.2 Antiviral drugs targeting m^6^A erasers

Specifically, m^6^A erasers play important roles in HIV infection, and their activation or inhibition exerts distinct effects at different stages of the viral life cycle. Activation of erasers has been reported to enhance antiviral immunity (Chen et al., 2021). In contrast, inhibition of FTO suppresses the packaging of full-length genomic RNA into viral particles, while simultaneously promoting Gag expression and p24 release, which enhances viral replication and concurrently reverses viral latency (Zhao et al., 2020; Tirumuru et al., 2016; Lichinchi et al., 2016a). Thus, given their alignment with the function of conventional latency-reversing agents (LRAs), inhibitors of m^6^A erasers may play a supplementary role in the “shock-and-kill” strategy. Ali et al. found that combining the ALKBH5 inhibitor ALKi-3 with the LRA romidepsin significantly enhanced viral reactivation, which also reduced off-target effects and toxicity by using a lower dosage of romidepsin (Ali et al., 2025; Zhao et al., 2019).

However, the functional impact of m^6^A erasers vary across different viruses. For example, inhibition of FTO promotes KSHV lytic replication, while suppression of ALKBH5 enhances PEDV (porcine epidemic diarrhea virus) infection but inhibits HBV replication (Jin et al., 2022; Tsukuda et al., 2024; Ye et al., 2017). Therefore, activation or inhibition of m^6^A erasers should be dependent on specific infection settings.

#### 4.1.3 Antiviral drugs targeting m^6^A readers

The small molecules Cucurbitacin B has been reported to suppress HBV replication both in vitro and in vivo by disrupting the interaction of IGF2BP1 with viral RNAs (Li et al., 2026). Other inhibitors targeting m^6^A readers such as YTHDF1 and YTHDC1, are still under development. Ebselen, an inhibitor of YTHDFs, has been reported to exert antiviral effects against SARS-CoV-2 and HCV, though its antiviral effects are not specifically attributed to the inhibition of m^6^A readers (Haritha et al., 2020; Mukherjee et al., 2014; Micaelli et al., 2022).

### 4.2 Antiviral drugs targeting viral molecular mechanisms hijacking the m^6^A machinery

While targeting the host m^6^A machinery offers potential antiviral benefits, it may induce off-target effects by disrupting global m^6^A distribution (Liu et al., 2024; Li et al., 2021). In contrast, targeting viral mechanisms that hijack host m^6^A pathways provide a more selective approach to disrupting viral replication while minimizing host toxicity.

**Fig. 3:**
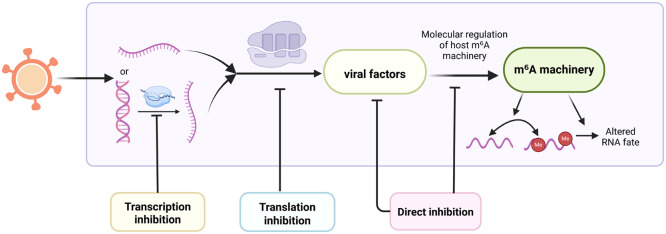
Antiviral strategies targeting the viral factor-m^6^A machinery axis. This schematic illustrates three mechanistic levels of antiviral intervention against viral factors that regulate host m^6^A machinery. Transcription inhibition suppresses viral gene expression before viral RNA production, translation inhibition blocks viral protein synthesis from viral RNA, and direct inhibition targets the activity or interaction of viral factors after their production. By acting at these distinct stages, these strategies may prevent viral factor-mediated remodeling of host m^6^A machinery, altered m^6^A marks and RNA fate of viral or host transcripts.

Targeting viral components that participating in hijacking m^6^A machinery, these antiviral drugs operate through several mechanisms. Direct inhibition interferes with viral components, transcription inhibition impairs the virus’s ability to transcribe its genome, and translation inhibition disrupts the translation of viral mRNA into proteins (Fig. 3). However, the majority of these drugs’ interaction with m^6^A pathway remain to be elucidated, and further investigation are needed to fully assess their therapeutic potential and safety. Representative antiviral strategies targeting viral factors implicated in the regulation or hijacking of host m^6^A machinery are summarized in Table 3 (Madruga et al., 2007; Ghosh et al., 2023; Cvetkovic and Goa, 2003; Vella and Saquinavir, 1998; Hammer et al., 1997; Musharrafieh et al., 2019; Welsch et al., 2008; Moerdyk-Schauwecker et al., 2009; Bellizzi et al., 2024; Gane et al., 2023; Yuen et al., 2024; Sekiba et al., 2019; Yuan et al., 2026; Corthésy et al., 2006; Caddy et al., 2020; Vega et al., 2013; Garaicoechea et al., 2008; Chen et al., 2004; Harmsen et al., 2007).

**Table 3:** Representative antiviral strategies targeting viral factors implicated in the regulation of host m^6^A machinery.

Targets of viral component	Drugs	Mechanism of Actions	Development status	Reference
HIV-1 Protease	HIV protease inhibitor: Lopinavir Saquinavir Indinavir Darunavir	Binds HIV-1 protease active site to block cleavage of Gag/Pol and prevent mature virion formation	FDA-approved for HIV treatment	Cvetkovic and Goa, 2003, Ghosh et al., 2023, Hammer et al., 1997, Madruga et al., 2007, Vella and Saquinavir, 1998
Enterovirus D68 2A Protease	Telaprevir	Primarily targets the HCV NS3/4A serine protease, but also inhibits EV-D68 2A protease	Preclinical repurposing candidate for EV-D68	Musharrafieh et al., 2019, Welsch et al., 2008
HSV ICP27	PPMO (Peptide-conjugated morpholino)	Binds complementarily to the translation-start-site region of ICP27 mRNA and suppresses ICP27 translation	Preclinical	Moerdyk-Schauwecker et al., 2009
	CRISPR-Cas9 targeting ICP27	Introduces site-specific double-strand breaks within the ICP27 (UL54) locus to disrupt ICP27 gene	Preclinical	Bellizzi et al., 2024
HBV HBx	VIR-2218 (GalNAc-conjugated siRNA)	Degrades all major HBV transcripts and reduces viral protein production through RNA interference (RNAi)-mediated strategy	Phase 2 clinical development for chronic HBV infection	Gane et al., 2023, Yuen et al., 2024
	Nitazoxanide (NTZ)	Disrupts the HBx–DDB1 interaction, which protects the Smc5/6 restriction complex and reduces cccDNA transcription	Preclinical	Sekiba et al., 2019
PoRV VP6	HBXIP-derived peptide (D-TK)	Disrupts the HSPB1-HBx interaction to suppress cccDNA	Preclinical	Yuan et al., 2026
	VP6-specific monoclonal antibody	Intracellular neutralization of rotavirus by binding VP6 on intracellular viral particles	Preclinical	Caddy et al., 2020, Corthésy et al., 2006
	VHH nanobodies against VP6	Intracellular neutralization of rotavirus by recognizing conserved conformational epitopes on VP6	Preclinical	Garaicoechea et al., 2008, Vega et al., 2013
FMDV VP1	siRNA against VP1	RNAi-mediated degradation of VP1 mRNA	Preclinical	Chen et al., 2004
	VHH nanobodies against VP1	Neutralization by binding VP1 GH-loop	Preclinical	Harmsen et al., 2007

## 5 Conclusion

N^6^-methyladenosine (m^6^A) modification represents a critical and dynamically regulated epitranscriptomic marks on RNA, governed by a sophisticated machinery comprising writers, erasers and readers. This modification exerts pivotal roles in modulating viral replication and host immune responses. Through post-translational modifications, proteolytic cleavage and selective recruitment of the m^6^A machinery, viruses reprogram the host epitranscriptome in a highly context-dependent manner. Such hijacking strategies underscore the central function of m^6^A in viral infection, highlight its essential role in viral pathogenesis, and position m^6^A as a promising target for antiviral intervention.

In recent years, the development of antiviral drugs has increasingly shifted toward precision-targeted therapeutic strategies. The molecular mechanisms by which viruses hijack the host m^6^A pathway provide valuable opportunities for the discovery of novel antiviral drugs. Despite the considerable therapeutic potential of targeting the m^6^A machinery, several key challenges should be considered. First, global perturbation of m^6^A regulation may cause widespread disruption of normal host cellular processes, thereby raising potential safety and toxicity concerns. In addition, the development of specific inhibitors against viral manipulation of the m^6^A machinery is still in the early stages. Most current drugs and small molecules remain in preclinical evaluation or experimental use, and their selectivity and long-term efficacy against viral infections require further systematic investigation.

Current researches indicate that non-structural viral proteins, including viral proteases, kinases and accessory factors, are far more frequently involved in hijacking the host m^6^A machinery than structural proteins, making them promising antiviral targets for disrupting viral exploitation of the m^6^A pathway. Advances in computer-aided drug design (CADD), structure-based drug screening, molecular docking, high-throughput virtual screening and artificial intelligence-driven modeling are accelerating the identification of specific antiviral small molecules. Further in-depth mechanistic studies of viral epitranscriptomic hijacking will be essential for the rational design and development of selective small-molecule inhibitors targeting viral manipulation of the m^6^A machinery.

## Consent for publication

All authors had access to the study data and have reviewed and approved the final manuscript.

## Funding

This work was supported by the Key R&D Program of Hubei Province [2021BCD004].

## CRediT authorship contribution statement

**Xinzhe Wu:** Writing – review & editing, Writing – original draft, Formal analysis, Data curation, Conceptualization. **Zhuoxuan Deng:** Validation, Data curation. **Yunkai Li:** Writing – original draft, Resources. **Chunwei Shi:** Writing – review & editing, Writing – original draft, Supervision, Project administration, Funding acquisition, Conceptualization.

## Declaration of competing interest

All authors have reviewed and approved the final manuscript. No conflict of interest exists.

## Data Availability

The data that has been used is confidential.
